# Supplementary material for: The efficacy of IL-6 inhibitor Tocilizumab in reducing severe COVID-19 mortality: a systematic review
Source: PeerJ. 2020 Nov 2;8:e10322. doi: 10.7717/peerj.10322 (PMC7643559; doi:10.7717/peerj.10322)
Supplement: Supplemental Information 1 [file peerj-08-10322-s001.docx]

**Systematic Review:**

- The two authors who performed the search are Avi Kaye and Dr. Robert Siegel
- Disagreements were to be resolved by consulting a third academic as outlined in the PRISMA registration (CRD42020193479). However, there was no disagreement that needed to be resolved between the two authors.

**Systematic Review Rationale:**

1. Without proven therapies or a vaccine for COVID-19, it is imperative that we investigate existing medications that can mitigate the effect of the disease. IL-6 inhibitor tocilizumab is currently under clinical trials for reduction of COVID-19 mortality. Furthermore, it is being used under emergency use authorization without proof of efficacy. This systematic review covers the rationale for IL-6 inhibitors and examines the existing evidence for whether tocilizumab reduces severe COVID-19 mortality to determine the scientific and ethical basis for ongoing clinical trials.
2. This systematic review is the most comprehensive and up to date examination of tocilizumab and its potential effect on reducing severe COVID-19 mortality. Past systematic reviews either only covered uncontrolled trials or a handful of controlled trials without quantitative synthesis.
