# Supplementary material for: The efficacy of IL-6 inhibitor Tocilizumab in reducing severe COVID-19 mortality: a systematic review
Source: PeerJ. 2020 Nov 2;8:e10322. doi: 10.7717/peerj.10322 (PMC7643559; doi:10.7717/peerj.10322)
Supplement: Supplemental Information 2 [file peerj-08-10322-s002.docx]

**Supplementary Table 1:** Study Characteristics for the Controlled Trials

| Study | Methods | TCZ Administration | SOC | Length of Observation |
| --- | --- | --- | --- | --- |
| Klopfenstein et al. | Retrospective case-control study of hospitalized COVID-19 patients, 20 of whom received TCZ and compared with 25 SOC patients in one French hospital. | 1 or 2 doses | Hydroxychloroquine or lopinavir/ritonavir and antibiotics, some corticosteroids | Until death and/or ICU admission |
| Campochiaro et al. | Claimed to be the first comparison of TCZ to SOC. Retrospective cohort study of 65 patients in one Italian hospital with severe COVID-19 outside the ICU. 32 were treated with TCZ and compared outcomes with standard of care after 28 days. | 2 doses [24 hrs. apart], 400 mg | Hydroxychloroquine, lopinavir/ritonavir, ceftriaxone, azithromycin, enoxaparin | 28 days |
| Capra et al. | Cohort study of 62 patients treated with TCZ within 4 days post-admission compared to 23 who received only SOC at one Italian hospital. Included severe, but pre-ICU or mechanical ventilation. | 1 dose, 400 mg IV or 324 mg s.c. | Lopinavir and ritonavir | Admission to April 2, 2020 |
| Colaneri et al. | Retrospective, observational analysis of 21 patients treated with TCZ and matched 1:1 to patients receiving standard of care (SOC) based on propensity score. Mortality assessed after 7 days. Performed at one facility in Italy. Included patients in the ICU. | 2 doses [12 hrs. apart], 8 mg/kg (up to 800 mg), | Hydroxychloroquine, azithromycin, heparin, methylprednisone | 7 days |
| Rojas-Marte et al. | Retrospective case-control study in a New York medical center. Included 193 patients with mild to critical COVID-19 comparing those who received TCZ against individuals who underwent SOC therapies. | Not specified | Hydroxychloroquine, and azithromycin. Some corticosteroids, anticoagulation, remdesivir, antibiotics and vasopressors. | Not specified |
| Wadud et al. | Retrospective case-control study of COVID-19 patients with ARDS comparing 44 treated with TCZ and 50 controls matched on age, sex, BMI and baseline inflammatory markers. Data derived from one medical facility in New York. | Not specified | Combinations of hydroxychloroquine, azithromycin and steroids | Until death or discharge |
| Ip et al. | Retrospective observational case-control study of tocilizumab administration for 547 ICU patients in a 13-hospital network in New Jersey. | 1 (n=104) or 2 doses (n=20) with 400mg followed by 800 mg, 8 mg/kg or 4 mg/kg | Not specified | 30 days |
| Roumier et al. | Prospective case-control study of TCZ treatment in 30 patients with severe COVID-19 matched 1:1 based on age, gender and disease severity. Performed in one facility in France. | 1 dose, 8mg/kg | Hydroxychloroquine and azithromycin | Median 8 days |
| Guaraldi et al. | Retrospective case-control analysis of multiple medical centers in Italy. Compared 179 patients who received TCZ against 365 individuals who were only given the SOC. Patients were randomly assigned for TCZ administration. | 2 doses [12 hrs. apart], 8 mg/kg IV or 162 mg s.c. | Hydroxychloroquine, azithromycin, antiretrovirals, heparin | Until death, discharge or mechanical ventilation |
| Patel et al. | Retrospective cohort study using data extracted Seattle medical center comparing 42 TCZ recipients matched with 41 controls. | Not specified | Not specified | 7 days |
| Eimer et al. | Retrospective cohort study in a Swedish medical center of ICU patients with 29 TCZ patients and 58 controls; 22 were matched 1:1. | 8 mg/kg | Not specified | 30 days |
| Canziani et al. | Retrospective case-control study in two Italian hospitals of 64 TCZ patients matched 1:1 with controls | 2 doses [24 hours], 8 mg/kg | Enoxaparin, lopinavir/ritonavir, hydroxychloroquine, antibiotics, glucocorticoids | 30 days |
| Gokhale et al. | Retrospective cohort study in an Indian medical center contrasting 70 patients who received TCZ against 91 who did not. | 1 dose, 400 mg | Antibiotics, hydroxychloroquine, ivermectin, oseltamivir, heparin, methylprednisolone | 6-week total observation period |
| Rossotti et al. | Retrospective analysis in a single Italian hospital of 77 TCZ-treated patients matched 1:2 with 148 SOC | 1-2 doses [12 hrs.], 8 mg/kg | Hydroxychloroquine, remdesivir, lopinavir/ritonavir | 40 days |
| Somers et al. | Prospective cohort study for ventilated COVID-19 patients in a Michigan hospital contrasting 78 TCZ patients against 76 SOC. | 1 dose, 8mg/kg (within 48 hours of intubation) | Combinations of hydroxychloroquine, remdesivir, corticosteroids and ACE inhibitors | 28 days, Median 67-day follow-up |
| Potere et al. | Retrospective case-control study in an Italian hospital comparing 40 TCZ+SOC patients against 40 SOC matched for sex and age. | 2 doses, 324 mg | Not specified | 35 days |

| Study | Adverse Effects | Duration of Hospitalization and % ICU Admission TCZ | Duration of Hospitalization and % ICU Admission SOC | Mean/Median Age TCZ | Mean/Median Age Control | TCZ Sex and Clinical Characteristics |
| --- | --- | --- | --- | --- | --- | --- |
| Klopfenstein et al. | None found | 13 days (mean)  5/20 (25%) | 17 days (mean)  18/25 (72%) | 76.8 (mean) | 70.7 (mean) | 45% male; higher comorbidity index and oxygen requirements than controls. Pre-ICU upon admission. |
| Campochiaro et al. | Bacterial/fungal infection in 13% TCZ and 12% SOC patients | 13.5 days (median)  4 (13%) – All survived | 14 days (median)  2 (6%) – One death | 65 (mean) | 60 (mean) | 91% male; 78% non-invasive ventilation, 22% high supplemental O2 |
| Capra et al. | No secondary infections reported | 12.5 days (mean)  17/62 (27%) | 8 days (mean)  23/23 (100%) | 63 (mean) | 70 (mean) | 73% male; oxygen requirements but not mechanical ventilation (MV) |
| Colaneri et al. | None found | Not reported  Odds ratio 0.11 (95% CI 0.00-3.38) | Not reported | 63.74 (median) | 62.33 (median) | 90% male; all patients in "severe" condition |
| Rojas-Marte et al. | Bacteremia less common in TCZ (13% versus 24%). Fever, cough and shortness of breath more common in TCZ. | 14.5 days (mean)  ICU admission not reported  Statistically significant lower mortality in non-ICU patients | 16.5 days (mean) | 60 (mean) | 60 (mean) | 77.1% male; 6.3% moderate (nasal canula), 30.2% very severe (high-flow O2/non-rebreather), 63.5% critical (intubated) |
| Wadud et al. | None reported | 17.9 days (mean) – Higher than SOC  Not reported | Not reported  Not reported | 55.5 (mean) | 66 (age) | Sex breakdown not included; statistically higher initial inflammatory markers than controls |
| Ip et al. | 18 (13%) of TCZ and 44 (11%) of controls had secondary bacteremia; 12 (9%) of TCZ and 25 (6%) of controls had secondary pneumonia | Not reported  All patients initially in ICU | Not reported | 62 (mean) | 60 (mean) | 28% male; all initially in ICU |
| Roumier et al. | Mild hepatic cytolysis (n=2) and ventilator-acquired pneumonia (n=1) | Not reported  Odds ratio 0.42 (95% CI 0.20-0.89) | Not reported | 62.3 (mean) | 60.6 (mean) | 80% male; 23.3% in ICU at baseline |
| Guaraldi et al. | One patient had an injection site reaction, 1 episode of neutropenia, 1 HSV1 reactivation causing liver sepsis | 12 days (mean)  Adjusted hazard ratio 0.61 (0.40-0.92) | 8 days (mean) | 64 (mean) | 69 (mean) | 71% male; O2 support required, no MV |
| Patel et al. | None reported | 29% still hospitalized  2 /21 (9.5%) | 49% still hospitalized  Value not provided. Reported as higher | 68 (median) | Not specified | 50% severe, 50% critical |
| Eimer et al. | None reported | Significantly shorter hospitalization  All patients admitted to ICU | Value not provided | 56 (median) | 56 (median) | ICU |
| Canziani et al. | Some bleeding, thrombosis and secondary infections | Not reported  Hazard ratio 0.36 (95% CI 0.16-0.83) | Not reported | 63 (mean) | 64 (mean) | 73% male |
| Gokhale et al. | None reported | 14 days (mean)  2/70 (2.9%) | 6 days (mean)  8/91 (8.8%) | 52 (median) | 55 (median) | 67.1% male; "severe," pre ventilation |
| Rossotti et al. | Increased hospital stay, 32.4% infectious complications mostly in ICU, 1 death from sepsis | Longer hospital stay  Not reported | Value not provided  Not reported | 59 (median) | 59 (median) | 82.4% male; severe, presence of respiratory distress |
| Somers et al. | Increased proportions of superinfections (56%), but no difference in 58-day mortality | 20.4 days (mean)  17/78 (22%) | 22.9 days (mean)  17/76 (22%) | 55 (mean) | 60 (mean) | 68% male; patients on MV |
| Potere et al. | No clinically serious adverse events, 2.5% bacterial pneumonia | Not reported  2/40 (5%) | Not reported  12/40 (30%) | 56 (mean) | 54.5 (mean) | 65% male; MV |
