# Supplementary material for: The efficacy of IL-6 inhibitor Tocilizumab in reducing severe COVID-19 mortality: a systematic review
Source: PeerJ. 2020 Nov 2;8:e10322. doi: 10.7717/peerj.10322 (PMC7643559; doi:10.7717/peerj.10322)
Supplement: Supplemental Information 3 [file peerj-08-10322-s003.docx]

**Supplementary Table 2:** Individual Strengths and Additional Shortcomings of Controlled Trials

| Study | Strengths | Shortcomings |
| --- | --- | --- |
| Klopfenstein et al. | Consistent inclusion/exclusion criteria for cases and controls. Analyzed both mortality and TCZ administration. | TCZ patients had higher comorbidity indices and worse baseline characteristics. Cases and controls not matched. Variation in SOC and number of TCZ doses. Cases and controls treated at different times. Concluded observation on April 24^th^ regardless of patient condition. |
| Campochiaro et al. | Cases and controls were performed at the same time. 28-day follow up. Provide longitudinal survival curve. | More adverse effects in ICU patients. SOC and TCZ not matched. TCZ patients admitted to ICU were given 2^nd^ dose. |
| Capra et al. | Consistent inclusion criteria and SOC. Attempted to address non-randomized assignment. | Cases and controls treated at different times. 2.7 times more TCZ patients than controls. No statistics on difference in baseline clinical characteristics between cases and controls. |
| Colaneri et al. | Cases and controls matched 1:1 based on propensity score. Patients all treated in 2-week period. Provide baseline and post-treatment biomarkers. | Only compare 7-day mortality rate. Missing clinical data reported. |
| Rojas-Marte et al. | Large sample size; similar between cases and controls. Controls had similar oxygen requirements. Primary endpoint of mortality. Compared intubated and non-intubated patients. All cases received one dose of TCZ. | Variation in disease severity; not addressed statistically. |
| Wadud et al. | Attempted to match cases with controls. Obtained data up until discharge or death. | TCZ patients had worse baseline clinical characteristics. Difference in SOC. Some missing clinical data for laboratory values. |
| Ip et al. | Large sample size. Consistent inclusion criteria (only evaluated ICU patients for cases and controls). 30-day observation period. | Analysis of TCZ was secondary in the study. Difference in SOC and TCZ administration between the 13 facilities. |
| Roumier et al. | Matched cases and controls 1:1. Analyzed ICU admission. | Some patients received other drugs in addition to TCZ. Median follow-up of 8 days. Controls treated at different time. |
| Guaraldi et al. | Large study size. Similar percentage of patients on ventilators. Same SOC for all patients. Provided longitudinal analysis and Kaplan-Meier. | Differences in TCZ administration. Variation in disease severity. Difference in glucocorticoid administration. TCZ groups had higher percentage of hypertension and diabetes. |
| Patel et al. | 1:1 case-control match. Analyzed multiple variables – like subsequent MV – to determine TCZ efficacy. Severe vs. critical illness analysis. | Retrospective matching of controls with cases, deemed an “informal” comparison. Short observation period. Patients received other therapies. |
| Eimer et al. | All patients began in ICU. Post hoc propensity score matching did not change results. TCZ and SOC group both consecutive patients. Long follow-up period. Analyzed ventilator-free days. | Cases and controls not matched. Low prevalence of comorbidities and median age. 1 facility; limited generalizability. Only focus on critical patients. |
| Canziani et al. | Multi-center with same protocol. Cases-controls matched 1:1. Long observation period. Patients included in national clinical trial. Determined mortality significantly associated with comorbidities. | Difference in hydroxychloroquine administration. Authors note many patients were potentially too compromised upon TCZ delivery for benefit. |
| Gokhale et al. | Consistent SOC. Multivariate analysis yielded statistically significant difference in mortality. Data from consecutive patients in 6-week period. | Controls not matched. TCZ younger median. One center. Broad inclusion criteria. |
| Rossotti et al. | 1:2 matching. Consecutive patients included. | Single center. |
| Somers et al. | Statistically significant difference after multivariate analysis/adjustment. Relatively long follow-up. All ventilated. | Single-center. TCZ patients younger and fewer comorbidities. Some patients transferred from outside hospitals. Only focus on MV. TCZ administration not standardized. |
| Potere et al. | Matching based on age and sex. Consistent TCZ administration. | One hospital. No info on SOC. Not matched based on severity. Selection process unclear. |
