# Supplementary material for: The efficacy of IL-6 inhibitor Tocilizumab in reducing severe COVID-19 mortality: a systematic review
Source: PeerJ. 2020 Nov 2;8:e10322. doi: 10.7717/peerj.10322 (PMC7643559; doi:10.7717/peerj.10322)
Supplement: Supplemental Information 4 [file peerj-08-10322-s004.docx]

**Supplementary Table 3:** Study Characteristics for the Uncontrolled Trials

| Study | Methods | TCZ Administration | Additional Treatments | Length of Observation | Mortality |
| --- | --- | --- | --- | --- | --- |
| Luo et al.^45^ | Retrospective analysis of 15 COVID-19 patients of varying severity treated with TCZ in a hospital in China. | 240-600 mg | Not specified | 7 days | 3/15 (20%) |
| Toniati et a.^46^ | Prospective trial of 100 consecutive patients comparing ICU (n=57) and non-ICU patient on noninvasive ventilation in multiple Italian hospitals. Assessed at 10 days. | 2 doses [12 hrs. apart], 8 mg/kg | Not specified | 10 days | 20/100 (20%) |
| Xu et al.^47^ | Uncontrolled trial of TCZ administration for 20 patients with severe or critical COVID-19 in a hospital in China | 1-2 doses [12 hrs. apart], 4-8 mg/kg to 800 mg | Lopinavir/ritonavir, IFN-α, ribavirin, glucocorticoid, methylprednisolone | 11 days | 0/20 (0%) |
| Price et al.^48^ | Observational, retrospective study of 153 consecutive patients who were provided TCZ in a Connecticut medical center. Severe and non-severe patients had similar survival. | 1 dose (2 for patients with high BMI), 8 mg/kg to 800 mg | Combinations of hydroxychloroquine and antivirals | 21-day observation period (mortality after 14 days) | 20/153 (13%) |
| Sciascia et al.^49^ | Prospective open, single-arm study of 63 hospitalized patients in an Italian hospital with severe COVID-19, followed up 1, 2, 7 and 14 days afterwards. | 1-2 (n=52) doses, 8 mg/kg IV or 324 mg s.c. | Not specified | 14 days | 7/63 (11.1%) |
| Alattar et al.^50^ | Retrospective review of 25 patients in Qatar with severe COVID-19 receiving TCZ, followed up 1, 3, 7 and 14 days later. 68% of patients had radiological improvement by day 14. | 1 dose, median 5.7 mg/kg | Hydroxychloroquine, azithromycin, lopinavir/ritonavir, ribavirin, interferon | 14 days | 3/25 (12%) |
| Issa et al.^51^ | Retrospective analysis following 10 patients who received TCZ at a hospital in Bordeaux, France. | 1 dose, 8 mg/kg | Hydroxychloroquine, ceftriaxone | 11 days | 1/10 (10%) |
| Quartuccio et al.^52^ | Retrospective study of hospitalized COVID-19 patients divided between 42 severe cases who received TCZ and 69 SOC patients. | 1 dose, 8 mg/kg | Methylprednisolone, antivirals (remdesivir), lopinavir/ritonavir, darunavir/cobicistat, hydroxychloroquine | Not specified | 7/41 (17.1%) |
| Campins et al.^53^ | Prospective study on TCZ administration for 58 people with severe COVID-19 in a Spanish medical facility. | Not specified | Corticosteroids | 28 days | 8/58 (13.8%) |
| Morena et al.^54^ | Open-label prospective study of 51 patients with severe COVID-19 in an Italian hospital receiving IV TCZ and tracked for at least 30 days or until death. | 2 doses, 400mg or 8 mg/kg [12 hrs. apart] | Hydroxychloroquine, and lopinavir-ritonavir | 34 days (median) | 14/51 (27.5%) |
| Borku Uysal et al.^55^ | Retrospective analysis of 12 patients pre-ICU who were given TCZ administration in Istanbul, Turkey. | 2 doses [24 hrs. apart], 400 mg | Hydroxychloroquine, oseltamivir, azithromycin or moxifloxacin | Until discharge or death | 0/12 (0%) |
| Strohbehn et al.^56^ | Phase II trial of 32 patients provided differing doses of TCZ | 2 doses, 200 mg, 120 mg, 80 mg or 40 mg | Not specified | 28 days | 5/32 (15.6%) |
| Patel, A. et al.^57^ | Retrospective study using data from a hospital in India | 1 dose 8 mg/kg up to 800 mg | Hydroxychloroquine, azithromycin, ceftriaxone, heparin | Up to 26 days | 5/20 (25%) |
| Tomasiewicz et al.^58^ | Retrospective study of 28 patients who receiving TCZ at 7 medical centers in Poland | 1-2 doses [8 hrs.], 800mg | Chloroquine and lopinavir/ritonavir | 14 days | 2/28 (7.1%) |
| Moreno-Pérez et al.^59^ | Retrospective cohort study of 77 patients treated with TCZ at a Spanish hospital. | 1-3 doses, 600mg for 1st and 400mg for 2nd or 3rd | Hydroxychloroquine, lopinavir/ritonavir, azithromycin | Median 86 days | 10/77 (12.9%) |
| Fernández‐Ruiz et al.^60^ | Retrospective cohort study of 88 consecutive patients who received TCZ in a Spanish medical center. | 1-4 doses [12 and 24 hours], 400 or 600mg then 400mg | Hydroxychloroquine, lopinavir/ritonavir, IFN-β and corticosteroids for some, antibiotics, heparin | 14 days | 6/88 (6.8%) |
| Knorr et al.^61^ | Retrospective analysis of TCZ-treated severe COVID-19 patients in a Philadelphia hospital | 1-3 doses, 8 mg/kg up to 800 mg | Glucocorticoids and hydroxychloroquine | 30 days | 28/66 (42.4%) |
| Jordan et al.^62^ | Prospective cohort study of 27 consecutive patients with severe COVID-19 who received TCZ at a Los Angeles hospital | 1 dose, 400mg | Azithromycin and hydroxychloroquine | Until discharge or death | 2/27 (7.4%) |

| Study | Adverse Effects | % ICU Admission | Mean/Median Age | Sex and Clinical Characteristics |
| --- | --- | --- | --- | --- |
| Luo et al. | None found | Not reported | 73 (median) | 80% male; 13% moderately ill, 40% seriously ill, 47% critically ill |
| Toniati et al. | 2 cases of septic shock | 13 (25%) | 62 (median) | 88% male; 43% in the ICU, other 57% had O2 demands |
| Xu et al. | None | No new admissions | 56.8 (mean) | 85.7% male; All 20 patients required O2 therapy, 5% non-invasive ventilation, 10% MV |
| Price et al. | Few adverse events. 4% had neutropenia, 3% experienced bacteremia. | 7/135 (5.2%) for non-severe cases | 64 (median) | 53% male; 90% severe |
| Sciascia et al. | None reported | 5/63 (7.9%) | 62 (mean) | 88.8% male; only included patients with pathological pulmonary involvement and/or abnormal inflammatory markers |
| Alattar et al. | 92% experienced at least one adverse event, but not necessarily from TCZ | 80% patients initially in ICU | 58 (median) | 92% male; 84% on ventilation upon initiation |
| Issa et al. | No adverse effects | All patients initially in ICU | 66 (median) | 100% male; all admitted to the ICU |
| Quartuccio et al. | Some bacterial superinfections | New ICU admissions not reported | 62.4 (mean) | 69.4% male; 57% of patients in the ICU before TCZ |
| Campins et al. | None reported | 19/58 (32.4%) | 60.6 (mean) | 72.4% male; severe, pre-ICU |
| Morena et al. | Most frequent were increased hepatic enzymes, thrombocytopenia and bacterial/fungal infections | 17/77 (33%) “worsening” condition | 60 (median) | 78.4% male; 88% on high-flow O2, 11.7% on invasive ventilation, 85% "severe," 12% "critical" |
| Borku Uysal et al. | None found | 2/12 (16.7%) | 65.83 (mean) | 50% male; Cough, fever, 67% dyspnea |
| Strohbehn et al. | None reported | ICU admission data not reported | 69 (mean) | 50% male; severe |
| Patel, A. et al. | No adverse effects | 11/20 (55%) | 54 (mean) | 85% male |
| Tomasiewicz et al. | Slight ALT increase, no serious effects | 3/23 (13%) | 60.7 (mean) | 67.9% male; oxygen dependency |
| Moreno-Pérez et al. | Serious bacteremia in 14.2% of patients | 42/88 (54.5%) | 62 (median) | 64.9% male; High level of care and O2 requirements at baseline |
| Fernández‐Ruiz et al. | No attributable adverse events | Roughly 20% | 46.8 (mean) | 65.9% male; oxygen support |
| Knorr et al. | Did not assess | 3/17 (17.6%) | 61 (median) | 62.1% male; 27.3% on MV |
| Jordan et al. | No serious adverse events | Most patients initially in ICU | 63 (median) | 85% male; 77.7% on MV |
