# Supplementary material for: The efficacy of IL-6 inhibitor Tocilizumab in reducing severe COVID-19 mortality: a systematic review
Source: PeerJ. 2020 Nov 2;8:e10322. doi: 10.7717/peerj.10322 (PMC7643559; doi:10.7717/peerj.10322)
Supplement: Supplemental Information 5 [file peerj-08-10322-s005.docx]

**Supplementary Table 4:** Additional Shortcomings of Uncontrolled Trials

| Study | Shortcomings |
| --- | --- |
| Luo et al. | Difference in number of TCZ doses. Excluded patients. 1-week observation. SOC not delineated. Monitor disease progress by laboratory markers. |
| Toniati et al. | Some patients received three doses. 10-day follow-up. 57% of patients were treated outside of the ICU due to a lack of bed availability. |
| Xu et al. | 1 patient inexplicably dropped from analysis. 3 patients given 2^nd^ dose. |
| Price et al. | Variation in SOC. |
| Sciascia et al. | SOC not included; data from multiple centers assessed. Mortality/clinical improvement was not a primary end-point. Variation in TCZ administration. |
| Alattar et al. | Difference in SOC (antiviral agents). Could not determine source of adverse events. Variation in TCZ doses. |
| Issa et al. | Very small sample. Does not describe selection criteria. |
| Quartuccio et al. | Primary goal of study was to identify laboratory features to predict CODI-19 severity. “Controls” for trial who did not receive TCZ were patients who did not need oxygen support at baseline (therefore, the study was considered uncontrolled and only TCZ patients were assessed). Variation in glucocorticoid administration. |
| Campins et al. | SOC not provided other than corticosteroids, which varied amongst patients and could confound results. Inclusion criteria not outlined. Limited data provided in report. |
| Morena et al. | Difference in TCZ administration and SOC. |
| Borku Uysal et al. | Very small sample. Does not delineate inclusion criteria. |
| Strohbehn et al. | Range of TCZ doses. Primary endpoint was fever resolution and CRP response. Small, single-center. High epidemiological risk factors. |
| 1. Patel et al. | Small sample size. Low median age. One hospital. |
| Tomasiewicz et al. | Participant selection process unclear. Short observation period. Different co-treatments and comorbidities. |
| Moreno-Pérez et al. | One center. Inconsistent baseline characteristics. Comorbidity independent mortality factor. |
| Fernández‐Ruiz et al. | Primary analysis after only 7 days. Restrictive inclusion criteria. No multivariate analysis. Young mean participant age. |
| Knorr et al. | Unclear recruitment and inclusion methods. Change in TCZ dosing, reduced to one dose max midway through. High rates of hypertension, diabetes and obesity. |
| Jordan et al. | Single center. Limited to one dose. Seven patients also enrolled in placebo-controlled remdesivir trial. Limited follow-up time. |
