# Supplementary material for: The efficacy of IL-6 inhibitor Tocilizumab in reducing severe COVID-19 mortality: a systematic review
Source: PeerJ. 2020 Nov 2;8:e10322. doi: 10.7717/peerj.10322 (PMC7643559; doi:10.7717/peerj.10322)
Supplement: Supplemental Information 6 [file peerj-08-10322-s006.doc]

| **1Section/topic** | **#** | **Checklist item** | **Reported on page #** |
| --- | --- | --- | --- |
| **TITLE** | | |  |
| Title | 1 | Identify the report as a systematic review, meta-analysis, or both. | 1: “The Efficacy of IL-6 Inhibitor Tocilizumab in Reducing Severe COVID-19 Mortality: **A Systematic Review”** |
| **ABSTRACT** | | |  |
| Structured summary | 2 | Provide a structured summary including, as applicable: background; objectives; data sources; study eligibility criteria, participants, and interventions; study appraisal and synthesis methods; results; limitations; conclusions and implications of key findings; systematic review registration number. | 1 |
| **INTRODUCTION** | | |  |
| Rationale | 3 | Describe the rationale for the review in the context of what is already known. | 2-4 |
| Objectives | 4 | Provide an explicit statement of questions being addressed with reference to participants, interventions, comparisons, outcomes, and study design (PICOS). | 4: “to determine whether the drug is potentially effective at reducing severe COVID-19-related mortality, thus corroborating the logic for continuing RCTs to evaluate the potential use of IL-6 inhibitors.” |
| **METHODS** | | |  |
| Protocol and registration | 5 | Indicate if a review protocol exists, if and where it can be accessed (e.g., Web address), and, if available, provide registration information including registration number. | 6  (PRISMA: CRD42020193479 |
| Eligibility criteria | 6 | Specify study characteristics (e.g., PICOS, length of follow-up) and report characteristics (e.g., years considered, language, publication status) used as criteria for eligibility, giving rationale. | 5: ”Papers with primary data for a case-control study comparing mortality rate from severe COVID-19 between TCZ and standard of care (SOC) were included for data synthesis.” |
| Information sources | 7 | Describe all information sources (e.g., databases with dates of coverage, contact with study authors to identify additional studies) in the search and date last searched. | 5: PubMed and SearchWorks |
| Search | 8 | Present full electronic search strategy for at least one database, including any limits used, such that it could be repeated. | 5: “the primary search terms were “COVID-19” or “SARS-CoV-2” and “tocilizumab.”” |
| Study selection | 9 | State the process for selecting studies (i.e., screening, eligibility, included in systematic review, and, if applicable, included in the meta-analysis). | 5: Articles utilized for the systematic review were selected from a PubMed search on August 4, 2020. Both authors screened and reviewed each paper…” |
| Data collection process | 10 | Describe method of data extraction from reports (e.g., piloted forms, independently, in duplicate) and any processes for obtaining and confirming data from investigators. | 5: “Data was independently extracted by the readers.” |
| Data items | 11 | List and define all variables for which data were sought (e.g., PICOS, funding sources) and any assumptions and simplifications made. | Table 1, Supplementary Tables 1 and 3. Include: Mortality rate for TCZ and standard of care (SOC), number of participants, TCZ administration, SOC, study characteristics, mean/median age, days of follow-up, days hospitalization, percent ICU admission, percent male. |
| Risk of bias in individual studies | 12 | Describe methods used for assessing risk of bias of individual studies (including specification of whether this was done at the study or outcome level), and how this information is to be used in any data synthesis. | Figure 5: Funnel Plot |
| Summary measures | 13 | State the principal summary measures (e.g., risk ratio, difference in means). | 5: “mortality rate for the TCZ and SOC group were calculated. In the controlled studies, the odds ratio (RR) of mortality from COVID-19 with TCZ versus the SOC was determined followed by the 95% confidence interval (CI) and p-value calculation.” |
| Synthesis of results | 14 | Describe the methods of handling data and combining results of studies, if done, including measures of consistency (e.g., I^2^) for each meta-analysis. | 5: “The data from the individual controlled studies were synthesized by a random effects meta-analysis calculation using MedCalc software “ |

Page 1 of 2

| **Section/topic** | **#** | **Checklist item** | **Reported on page #** |
| --- | --- | --- | --- |
| Risk of bias across studies | 15 | Specify any assessment of risk of bias that may affect the cumulative evidence (e.g., publication bias, selective reporting within studies). | 8-9: “There are numerous notable methodological limitations in the studies analyzed including the lack of randomization in controlled trials and potential for an inadequate evaluation due to unpublished data.” |
| Additional analyses | 16 | Describe methods of additional analyses (e.g., sensitivity or subgroup analyses, meta-regression), if done, indicating which were pre-specified. | N/A |
| **RESULTS** | | |  |
| Study selection | 17 | Give numbers of studies screened, assessed for eligibility, and included in the review, with reasons for exclusions at each stage, ideally with a flow diagram. | Figure 1 |
| Study characteristics | 18 | For each study, present characteristics for which data were extracted (e.g., study size, PICOS, follow-up period) and provide the citations. | Table 1, Supplementary Tables 1 and 3 |
| Risk of bias within studies | 19 | Present data on risk of bias of each study and, if available, any outcome level assessment (see item 12). | Figure 5 |
| Results of individual studies | 20 | For all outcomes considered (benefits or harms), present, for each study: (a) simple summary data for each intervention group (b) effect estimates and confidence intervals, ideally with a forest plot. | Figure 4 |
| Synthesis of results | 21 | Present results of each meta-analysis done, including confidence intervals and measures of consistency. | Table 1 and Figure 4 |
| Risk of bias across studies | 22 | Present results of any assessment of risk of bias across studies (see Item 15). | Figure 5 |
| Additional analysis | 23 | Give results of additional analyses, if done (e.g., sensitivity or subgroup analyses, meta-regression [see Item 16]). | N/A |
| **DISCUSSION** | | |  |
| Summary of evidence | 24 | Summarize the main findings including the strength of evidence for each main outcome; consider their relevance to key groups (e.g., healthcare providers, users, and policy makers). | 7-8 |
| Limitations | 25 | Discuss limitations at study and outcome level (e.g., risk of bias), and at review-level (e.g., incomplete retrieval of identified research, reporting bias). | 8-9 |
| Conclusions | 26 | Provide a general interpretation of the results in the context of other evidence, and implications for future research. | 9: “the results from this systematic analysis corroborate the logic and ethical basis for ongoing phase III RCTs on TCZ.” |
| **FUNDING** | | |  |
| Funding | 27 | Describe sources of funding for the systematic review and other support (e.g., supply of data); role of funders for the systematic review. | No Funding |

*From:*  Moher D, Liberati A, Tetzlaff J, Altman DG, The PRISMA Group (2009). Preferred Reporting Items for Systematic Reviews and Meta-Analyses: The PRISMA Statement. PLoS Med 6(7): e1000097. doi:10.1371/journal.pmed1000097

For more information, visit: **www.prisma-statement.org**.

Page 2 of 2
